# Elucidating Structure Formation in Highly Oriented Triple Cation Perovskite Films

**DOI:** 10.1002/advs.202206325

**Published:** 2023-04-20

**Authors:** Oscar Telschow, Niels Scheffczyk, Alexander Hinderhofer, Lena Merten, Ekaterina Kneschaurek, Florian Bertram, Qi Zhou, Markus Löffler, Frank Schreiber, Fabian Paulus, Yana Vaynzof

**Affiliations:** ^1^ Integrated Center for Applied Physics and Photonic Materials Technische Universität Dresden Nöthnitzer Straße 61 01187 Dresden Germany; ^2^ Center for Advancing Electronics Dresden (cfaed) Technische Universität Dresden Helmholtzstraße 18 01069 Dresden Germany; ^3^ Institut für Angewandte Physik Universität Tübingen 72076 Tübingen Germany; ^4^ Deutsches Elektronen‐Synchrotron DESY Notkestr. 85 22607 Hamburg Germany; ^5^ Dresden Center for Nanoanalysis (DCN) Technische Universität Dresden Helmholtzstraße 18 01069 Dresden Germany

**Keywords:** antisolvent, crystalline structure, metal halide perovskites, orientation

## Abstract

Metal halide perovskites are an emerging class of crystalline semiconductors of great interest for application in optoelectronics. Their properties are dictated not only by their composition, but also by their crystalline structure and microstructure. While significant efforts are dedicated to the development of strategies for microstructural control, significantly less is known about the processes that govern the formation of their crystalline structure in thin films, in particular in the context of crystalline orientation. This work investigates the formation of highly oriented triple cation perovskite films fabricated by utilizing a range of alcohols as an antisolvent. Examining the film formation by in situ grazing‐incidence wide‐angle X‐ray scattering reveals the presence of a short‐lived highly oriented crystalline intermediate, which is identified as FAI‐PbI_2_‐xDMSO. The intermediate phase templates the crystallization of the perovskite layer, resulting in highly oriented perovskite layers. The formation of this dimethylsulfoxide (DMSO) containing intermediate is triggered by the selective removal of *N,N*‐dimethylformamide (DMF) when alcohols are used as an antisolvent, consequently leading to differing degrees of orientation depending on the antisolvent properties. Finally, this work demonstrates that photovoltaic devices fabricated from the highly oriented films, are superior to those with a random polycrystalline structure in terms of both performance and stability.

## Graphical abstract

In situ grazing‐incidence wide‐angle X‐ray scattering experiments elucidate that the structure of highly oriented triple cation perovskite films is templated by a highly ordered FAI‐PbI_2_‐xDMSO intermediate. The formation of this intermediate is triggered by the differences in the interaction of alcoholic antisolvents with the host solvents of the perovskite precursor solution. Photovoltaic devices fabricated with highly oriented films lead to better device performance and stability.

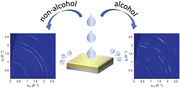

## 1 Introduction

Metal halide perovskites are a remarkable class of semiconductors whose excellent optoelectronic properties make them particularly promising for application in photovoltaics.^[^ ^1^, ^2^ ^]^ Over the last decade, significant advances have been made in the design of their composition,^[^ ^3^ ^]^ passivation of defects,^[^ ^4^ ^]^ interfacial engineering,^[^ ^5^ ^]^ and control over the layer's microstructure.^[^ ^6^ ^]^ The latter has been shown to be highly important, since the microstructure of the perovskite active layer has a significant effect not only on the optoelectronic properties and device efficiency,^[^ ^7^ ^]^ but also on the stability of perovskite solar cells.^[^ ^8^, ^9^ ^]^

Many different strategies have been employed for controlling the microstructure of the perovskite layer. For example, increasing the precursor concentration of the perovskite solution has been shown to lead to an increase in the grain size of the perovskite layer.^[^ ^10^, ^11^ ^]^ Alternatively, Lee et al. demonstrated that the microstructure of the perovskite active layer evolves when chlorine‐containing precursors such as lead chloride (PbCl_2_) or methylammonium chloride (MACl) are added to the perovskite solution, leading to significantly larger grain sizes.^[^ ^12^ ^]^ Moreover, the use of additives also proved effective in controlling the microstructure of perovskite layers. Notable examples of such additives are thiourea,^[^ ^13^ ^]^ ammonium hypophosphite (NH_4_H_2_PO_2_),^[^ ^14^ ^]^ and hypophosphorous acid.^[^ ^15^ ^]^ Finally, the surface properties of the substrate on top of which the perovskite precursor solution is deposited can also impact on the resultant microstructure. Nonwetting surfaces have been shown to lead to the formation of larger grains,^[^ ^16^ ^]^ but on the other hand might also result in microstructural defects such as pin‐holes and nanovoids.^[^ ^17^ ^]^

In addition to the size of the perovskite grains, recent studies suggest that their relative orientation with respect to the substrate and each other might impact the photovoltaic performance.^[^ ^18^ ^]^ For example, Yang et al. reported that the addition of caffeine into the perovskite solution results in a preferential orientation of the perovskite grains along the (110) planes. The authors suggest that this preferential orientation improves charge transport in the device, leading to enhanced photovoltaic performance.^[^ ^19^ ^]^ However, the impact of grain orientation could not be disentangled in this case, since the addition of caffeine also led to an increase in grain size and better defect passivation, which also result in improved device performance. Interestingly, the work examining the impact of chlorine also reported a preferential orientation along the (110) upon the addition of MACl into the perovskite precursor solution.^[^ ^12^ ^]^ Yet, also in this case, the impact of grain orientation could not be decoupled from the change in microstructure. On the other hand, spectroscopic studies suggest that neither the size, nor the orientation of perovskite grains impacts their optoelectronic properties,^[^ ^20^ ^]^ leaving the question of the consequences of crystalline orientation for device performance unanswered.

Importantly, the formation of perovskite films occurs via crystalline intermediate phases, often containing high boiling point solvent molecules in the crystal lattice.^[^ ^21^ ^]^ These intermediate phases convert to the photoactive perovskite phase upon thermal annealing.^[^ ^22^, ^23^ ^]^ Understanding the formation mechanisms of such intermediates, and the development of strategies to control them can enable precise structural engineering of the deposited perovskite layers.^[^ ^24^ ^]^ Among the most effective methods to investigate the temporal evolution of the crystallization process is by in situ grazing‐incidence wide‐angle X‐ray scattering (GIWAXS) measurements.^[^ ^25^, ^26^, ^27^ ^]^ Indeed, such characterization—although experimentally complex—has already led to significant insights. For example, Qin et al. identified three clear stages of film formation of metal halide perovskites, and demonstrated that annealing has to take place in the second stage, in order to avoid the formation of undesirable phases.^[^ ^28^ ^]^ Huang and co‐workers employed in situ X‐ray diffraction (XRD) to investigate the crystallization processes in FAPbI_3_ and demonstrated the presence of multiple solvent‐coordinated intermediate phases.^[^ ^29^ ^]^ While these examples illustrate the efficacy of in situ characterization for the study of perovskite crystallization processes, to the best of our knowledge, these techniques were not yet applied to the study of orientation control.

To examine the impact of orientation on the photovoltaic performance, it is thus important to not only isolate the orientational variation from microstructural changes, but also investigate the temporal evolution of structure formation, thus elucidating the mechanism that triggers orientational preference. In our previous work, we observed that the former can be made possible in case of perovskite layers fabricated via the antisolvent engineering route.^[^ ^30^ ^]^ Specifically, we observed that the use of alcohols as antisolvent leads to highly oriented films, while other antisolvents largely lead to a random grain orientation. A similar observation was later reported by Wang et al, who observed preferred orientation of perovskite layers fabricated using isobutanol (IBA) as an antisolvent.^[^ ^31^ ^]^ The authors suggested that the polarity of the antisolvent molecule led to a different orientation of formamidinium (FA^+^) molecules in an IBA‐DMSO‐FA^+^ complex as compared to the DMSO‐FA^+^ complexes formed when using a non‐alcoholic antisolvent. Importantly, the authors observed an improved photovoltaic performance for the oriented perovskite layers. While these results are highly promising, many questions regarding the structure formation of oriented perovskite films and the impact on the photovoltaic performance remain open. For example, it remains unclear which characteristics of the alcoholic antisolvents impact the orientation of the perovskite layers and how the relative degrees of orientation impact the performance and stability of perovskite solar cells.

**Figure 1:**
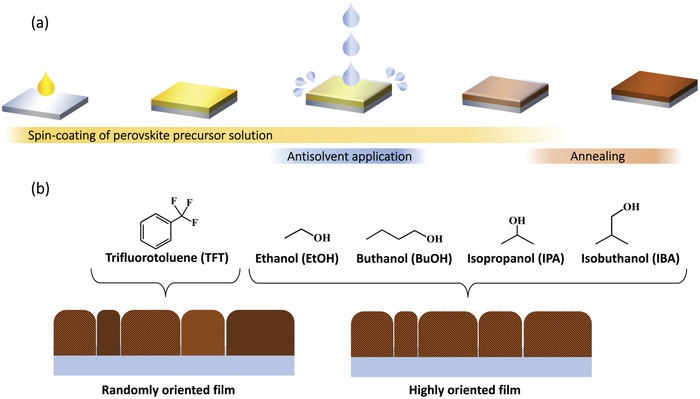
a) Schematic illustration of the antisolvent engineering method for perovskite layer deposition. b) Chemical structures and illustration of the corresponding film orientation of the antisolvents used in this study.

To address these questions, we investigate the temporal evolution of crystallization in triple cation perovskite films deposited by the antisolvent engineering method. In short, in this method the perovskite thin film is formed by spin‐coating the perovskite solution (in a 4:1 mixture of *N,N*‐dimethylformamide (DMF) and dimethylsulfoxide (DMSO)) on the substrate, during which an antisolvent is dripped onto the wet film, triggering crystallization. Once the spin‐coating procedure ends, the crystallization is completed by thermal annealing (**Figure** **1**a). To probe the structure formation, we employed in situ grazing‐incidence wide‐angle X‐ray scattering (GIWAXS) performed during the fabrication of the perovskite layers. Such in situ techniques proved to be highly effective in studying the crystallization processes of perovskite films, revealing both crystallization kinetics and growth mechanisms.^[^ ^32^ ^]^ We examine the structure formation of perovskite films fabricated using three different alcoholic antisolvents and compare it to films fabricated using a nonalcoholic solvent. The chemical structures of the examined antisolvents, namely butanol (BuOH), isopropanol (IPA), isobutanol (IBA), and trifluorotoluene (TFT) are shown in Figure 1b. Our measurements reveal the presence of a short‐lived, highly oriented intermediate species that templates the growth of the oriented perovskite layer. Finally, we compare the performance and stability of the fabricated perovskite solar cells, revealing that both these factors are correlated with the degree of crystal grain orientation.

## 2 Results and Discussion

### 2.1 Microstructure Characterization

**Figure 2:**
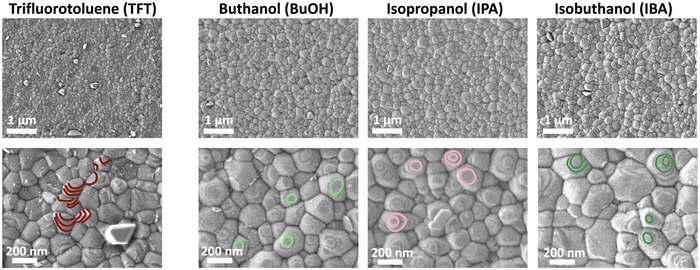
Scanning electron microscopy images collected via the secondary electron detector of triple cation perovskite films fabricated using different antisolvents. Exemplary grain edges have been highlighted to show the difference in orientation.

In our previous work we reported that the use of alcohols as antisolvents may lead to microstructural defects due to the extraction of organic halides during the antisolvent application step.^[^ ^30^ ^]^ Specifically, this may occur if the antisolvent is extruded slowly and the interaction time of the antisolvent with the spinning substrate and thinned precursor solution is not short enough. To avoid this, all films in this study were fabricated by extruding the antisolvents rapidly. To ensure no microstructural defects were formed, the films were examined using scanning electron microscopy (SEM). SEM images confirmed that polycrystalline and pinhole‐free perovskite films were fabricated using each of the antisolvents (**Figure** **2**). This has been further corroborated via cross‐sectional SEM imaging that confirmed that all antisolvents led to the formation of compact perovskite layers without any pin‐holes or nanovoids at the buried interfaces (Figure S1, Supporting Information). The grain size is similar in all the films with grains ranging from 50 to 300 nm in diameter. Interestingly, the change in the relative orientation in these films can be observed already via SEM. While grains in perovskite films fabricated using TFT exhibit various edges of crystal facets, grains on the IPA, IBA, and BuOH films often display a flat surface facing upward with concentric edges around them, suggesting crystal planes parallel to the film surface. Some of these edges have been highlighted in the high magnification images shown in Figure 2 for clarity.

### 2.2 Structural Characterization

To examine in detail the evolution of the crystalline structure during the formation of the films, in situ GIWAXS characterization was performed on a bespoke setup in which a spin‐coater was integrated into the synchrotron beamline and the reciprocal space maps were recorded as a function of time for each of the investigated antisolvents. The evolution of these maps as videos can be found as Note S1 (Supporting Information).

**Figure 3:**
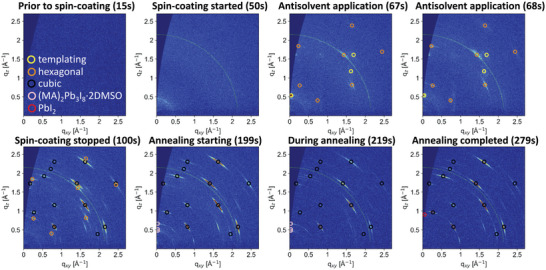
Grazing‐incidence wide‐angle X‐ray scattering (GIWAXS) data taken during different time points during film formation using isopropanol (IPA) as an antisolvent. The evolution for the other antisolvents can be seen in Figures S2–S4 (Supporting Information).

In **Figure** **3**, we present the GIWAXS maps at important time points during the deposition procedure for samples with IPA as an antisolvent, which will allow us to track the evolution of different structures formed during the film formation. At the first frame (15 s), taken after the perovskite precursor solution was dispensed, but prior to the start of spin‐coating, no crystalline features are observed. The spin‐coating procedure was started at 37 s, and shortly afterwards (50 s), the solution is thinned down, making it possible to observe the reflections associated with the indium tin oxide (ITO) substrate, which is marked in a dashed line (*q* = 2.15 Å^−1^). We note that the feature at 0.49 Å^−1^ originates from the Kapton window of the experimental setup and is present on all images independent from the sample properties. The antisolvent is dispensed at 67 s, at which point immediately two crystalline species can be observed. The first, marked in yellow circles, leads to a strong signal at *q*_z_ = 0.54 Å^−1^, which we assign to a highly oriented intermediate phase that templates the oriented growth of the perovskite, since—as will be shown in the following—it is only observed in the case of the alcoholic antisolvents. The intermediate species is very short lived and is observed only for 2 s under the applied preparation methods. The second species, marked in orange circles is a hexagonal phase of the triple cation perovskite.^[^ ^33^ ^]^ It is noteworthy that already at this stage the hexagonal phase exhibits a clearly preferred orientation, since distinct diffraction features are observed, rather than full diffraction rings. Shortly after the spin‐coating has finished (100 s), we observe a coexistence of the hexagonal and cubic phases (marked in black circles) of the perovskite layer.^[^ ^34^ ^]^ The latter also exhibits a highly oriented structure, evidenced by distinct diffraction features. Due to instrumental limitations, annealing could only commence roughly 2 min after the completion of spin‐coating. Approximately 100 s after the spin coating stopped (199s), we no longer observe a hexagonal phase of the perovskite, but instead detect the formation of a known (MA)_2_Pb_3_I_8_·2DMSO intermediate (pink circles).^[^ ^35^ ^]^ This intermediate remains for the first 20 s of annealing, but is eliminated after 90 s of annealing, at which point a small contribution associated with phase separated PbI_2_ can be observed (red circle) alongside highly oriented features of cubic perovskite.

**Figure 4:**
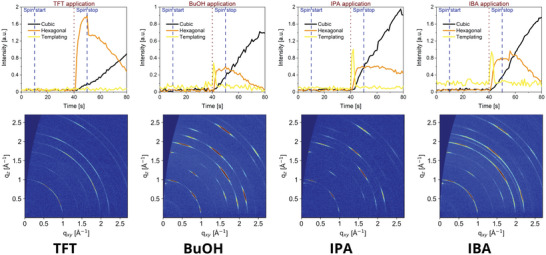
Temporal evolution of the intensity of the templating species, the hexagonal and cubic perovskite phases, and the final grazing‐incidence wide‐angle X‐ray scattering (GIWAXS) map obtained postannealing for each the investigated antisolvents by integrating the relevant diffraction rings for each of the tracked species.

To compare the structure evolution for the different antisolvents, we focus our attention on the templating species and hexagonal and cubic phases of the perovskite. **Figure** **4** displays the intensity evolution of these three species as a function of time and the final GIWAXS maps obtained postannealing for each of the films. In case of the TFT antisolvent, no templating species is observed and the formation of the hexagonal perovskite phase—which, in contrast to the alcohols, shows significantly less orientation—occurs once the antisolvent is dispensed. After an initial increase, this phase is decreased with an increasing intensity of the cubic phase. Once annealed, only cubic phase features remain, with a largely random orientation, evidenced by the Debye ring shape of the GIWAXS pattern. On the other hand, in the case of all three of the alcoholic antisolvents, a short‐lived templating species is observed immediately once the antisolvent is dispensed, which in all cases appeared at *q*_z_ = 0.54 Å^−1^. This finding suggests that the structure of this species is independent of the specific alcohol used, which implies that the antisolvent is not incorporated into that crystalline structure.

**Figure 5:**
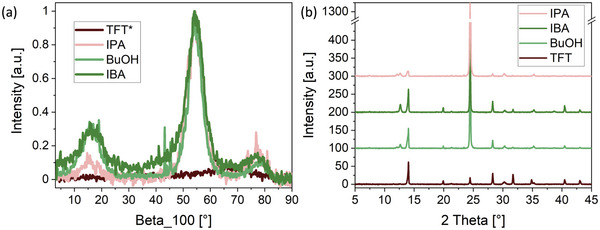
a) Angular profile along the (100) reflection of the grazing‐incidence wide‐angle X‐ray scattering (GIWAXS) maps shown in Figure 4. b) X‐ray diffraction (XRD) measurements on perovskite samples fabricated using different antisolvents. * For clarity of the graph, the trifluorotoluene (TFT) curve was normalized to the highest peak of butanol (BuOH) instead of its own highest peak.

To compare the degrees of orientation between the different samples, we performed angular integration along the (100) reflection as well as X‐ray diffraction (XRD) measurements that enable us to compare the intensity of the (111) reflections (*q* = 1.72 Å^−1^, 2*Θ* = 24.46°). The angular profiles confirm that no preferential orientation occurs in the case of the TFT samples, but a clearly preferred orientation is evident for all the alcoholic antisolvents with increased intensity at approximately 15°, 55°, and 77° (**Figure** **5**a). The distribution of intensities shows a dependency on the choice of antisolvent, with IPA leading to particularly oriented films with the strongest intensity at 55° in comparison to that at 15° and 77°. Similar observations can be made by examining the XRD patterns (Figure 5b). Very clearly, the (111) intensity is strongest in the IPA fabricated samples, although it is very prominent also in the other samples fabricated with alcoholic antisolvents.

To investigate whether there are differences in the vertical distribution of the crystalline species in the fully fabricated, annealed perovskite layers, we performed angular dependent GIWAXS measurements. At this stage, the films consist almost exclusively of the cubic perovskite phase and PbI_2,_ the vertical evolution of which is shown in Figure S5 (Supporting Information). The results reveal that the vertical distribution of the PbI_2_ is impacted by the choice of antisolvent: while alcoholic antisolvents lead to increased amounts of PbI_2_ in the bulk of the films. In the films made using TFT, its distribution is homogenous throughout the layers. In the past, we and others reported that eliminating PbI_2_ from the sample surface of triple cation perovskites leads to improved performance, in particular due to an increase in the device open‐circuit voltage (*V*_OC_).^[^ ^36^, ^37^, ^38^ ^]^ The potential impact of the differences in the PbI_2_ distribution on the device performance will be discussed in Section 2.4.

### 2.3 Proposed Mechanism for Structure Formation

As mentioned above, our previous observation that the short‐lived templating structure appears at *q*_z_ = 0.54 Å^−1^ regardless of the type of alcohol used suggests that the alcoholic antisolvent is not integrated into this crystalline structure, indicating that it consists of the precursors and/or solvents present in the wet perovskite film being spin‐coated. Its reflections do not coincide with the previously reported solvent complexes involving DMF or PbI_2_‐DMSO.^[^ ^32^, ^33^, ^34^, ^35^, ^36^, ^37^, ^38^, ^39^, ^40^, ^41^ ^]^ To gain further insights into the species that are integrated into the short‐lived intermediate phase, we examined the structure formation in MAPbI_3_ and FAPbI_3_ films fabricated with IPA as an antisolvent. Interestingly, GIWAXS measurements revealed that only the latter composition exhibited a templating structure (Figure S6, Supporting Information). This observation suggests that FA molecules are incorporated into the templating structure, since the high degree of orientation depends significantly on the FA content. Drop‐casting a highly concentrated solution of FAI and PbI_2_ in a molar 1:1 ratio in pure anhydrous DMSO resulted after gentle drying at 60 °C in a pale yellow, crystalline film that exhibits a series of intense reflections that are in a good agreement with those observed by GIWAXS for the templating structure (see Figure S7, Supporting Information). The crystalline film is highly ordered, which can also be observed via optical microscopy (Figure S8, Supporting Information). We note that single crystal structure characterization and in‐plane diffraction experiments failed due to the high sensitivity of these crystals, that converted to brown perovskites rather rapidly under light or X‐ray exposure, which is typical for perovskite intermediates that incorporate solvent molecules. Our experiments suggest a composition of FAI‐PbI_2_‐x·DMSO considering the 1:1 FAI to PbI_2_ ratio we used. Examining the literature reveals that an intermediate with this composition has been proposed by Ren et al,^[^ ^42^ ^]^ yet its complete crystalline structure has not been reported by the authors. The absence of DMF in the drop‐casting experiments proves that solely DMSO is incorporated into the crystal lattice of the observed intermediate upon treatment with alcoholic antisolvents. This suggests that alcoholic antisolvents are preferentially removing DMF from the DMF:DMSO host solvent mixtures used in the film fabrication. This hypothesis is supported by considering the Hansen solubility parameters of the solvents involved in the film fabrication process. Hansen solubility parameters, established by Charles M. Hansen in 1967,^[^ ^43^ ^]^ are defined as follows:

Δ*D* – The energy from dispersion forces between molecules
Δ*P* – The energy from dipolar intermolecular force between molecules
Δ*H* – The energy from hydrogen bonds between molecules.

Hansen defined the “Hansen space” as the three‐dimensional coordinate space (Δ*D*, Δ*P*, Δ*H*). The closer two molecules are to each other in this Hansen space, the more likely it is that they are capable of dissolving in each other. **Table** **1** lists the Hansen solubility parameters for the host solvents (DMF and DMSO) and the four antisolvents used in this study. Based on these parameters, we can calculate the distance between the corresponding coordinates in the Hansen space for each of the antisolvents with respect to the host solvents, defined as *R*_A_(DMF) and *R*_A_(DMSO). By examining these distances, we observe that the interaction of DMF with the alcoholic antisolvents is far stronger to that of DMSO, evidenced by the smaller values of *R*_A_(DMF). This is due to the stronger interaction via hydrogen bonds that can form between the alcohols and the polar DMF host solvent.

**Table 1:** Hansen parameters of the perovskite solvents and antisolvents used in this study

Solvent	Δ*D*	Δ*P*	Δ*H*	*R*_A_(DMF)	*R*_A_(DMSO)	*R*_A_(DMF)/*R*_A_(DMSO)
IPA	15.8	6.1	16.4	9.7	13.10	0.74
BuOH	16	5.7	15.8	9.6	13	0.74
IBA	15.1	5.7	15.9	10.31	13.8	0.75
TFT	17.5	8.8	0	12.32	12.85	0.96
DMF	17.4	13.7	11.3	0	3.54	–
DMSO	18.4	16.4	10.2	3.54	0	–

On the other hand, due to the absence of a hydroxyl group in TFT, only the dispersive and dipolar interactions determine the interaction among the solvents. These interactions are very similar for both host solvents, resulting in an equally good extraction of both DMF and DMSO by TFT. This is further illustrated by calculating the *R*_A_(DMF)/*R*_A_(DMSO) ratio, which is significantly smaller for the alcohols than for TFT. This difference in solvent interaction suggests that alcohols preferentially extract DMF from the precursor solution as it is more soluble in them, resulting in a local enrichment of DMSO on the substrate during the antisolvent treatment. The high DMSO concentration, in turn, enables the formation of the highly oriented FAI‐PbI_2_‐xDMSO intermediate that templates the crystallization of the perovskite and consequently its orientation. Employing TFT—or other nonalcoholic solvents—as antisolvent does not lead to a DMSO enriched environment and the DMSO‐intermediate does not form, thus leading to a lack of preferred orientation in the final perovskite film.

Taken together with the results of our previous studies,^[^ ^30^, ^44^ ^]^ the proposed mechanism adds an additional consideration into the selection of an antisolvent for perovskite film fabrication, resulting in three different factors that impact film formation:

1) The solubility of the perovskite precursors in the antisolvent: in case the chosen antisolvent can easily dissolve some of the perovskite precursors, its application may lead to an irreparable alternation of the intended film stoichiometry. This can be largely avoided by applying the antisolvent very fast,^[^ ^30^ ^]^ or by modifying its deposition strategy from pipetting to spraying.^[^ ^38^ ^]^
2) The miscibility of the antisolvent with the host solvents: certain antisolvents exhibit a very poor miscibility with the host solvents DMF and DMSO. In this case, the extraction of the host solvents is inefficient, often resulting in an incomplete film coverage.^[^ ^30^, ^44^ ^]^ To circumvent this issue, the antisolvent should be applied slowly in order to prolong the time during which the host antisolvents can be extracted.
3) The solubility of the antisolvent with the host solvents: considering that many perovskite compositions rely on the use of host solvent mixture (e.g., DMF and DMSO), the individual interactions of the antisolvent with each of the host solvents must be considered. Differences in the solubility of the antisolvent with each of the host solvents may lead to a preferential extraction of one over the other, which in turn can impact on the formation of solvent‐containing intermediate phases that guide perovskite crystallization.

These three factors have to be considered together when selecting the antisolvent, and are influenced by the specific perovskite composition, the desired stoichiometry, microstructure, and orientation. Moreover, the considerations outlined above can be used to choose a mixture of antisolvents that would lead to the desired film formation processes. For example, we fabricated perovskite layers using a 1:1 mixture of TFT and IPA. Such a sample exhibits preferential orientation as the one fabricated by IPA (Figure S9, Supporting Information), but the presence of TFT lowers the solubility of the perovskite precursors in the antisolvent mixture, thus relaxing the need to apply it very fast. Importantly, the use of antisolvent mixtures also opens the possibility to utilize them as a mean to incorporate additives or passivation agents to the perovskite surface.^[^ ^17^, ^45^ ^]^

### 2.4 Photovoltaic Characterization

**Figure 6:**
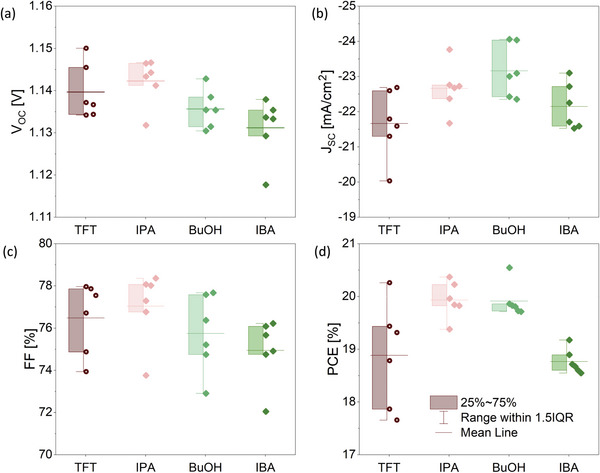
a) *V*_OC_, b) *J*_SC_, c) fill factor (FF), and d) power conversion efficiency (PCE) of photovoltaic devices fabricated using different antisolvents.

To investigate the performance of perovskite layers fabricated with the different antisolvents, we fabricated solar cells in an inverted architecture, with the structure glass/ITO/MeO‐2PACz /perovskite/PCBM/BCP/Ag (MeO‐2PACz: [2‐(3,6‐Dimethoxy‐9H‐carbazol‐9‐yl)ethyl]phosphonic acid; PCBM: [6,6]‐Phenyl‐C61‐butyric acid methyl ester; BCP: 2,9‐Dimethyl‐4,7‐diphenyl‐1,10‐phenanthrolin (bathocuproin)). The photovoltaic parameters of the best six solar cells of each kind are presented in **Figure** **6**. The *V*_OC_ and fill factor (FF) of the devices are very similar, but the short‐circuit current (*J*_SC_) shows clear difference, with devices fabricated with TFT as antisolvent yielding the lowest average photocurrent. This observation is in agreement with external quantum efficiency (EQE) measurements that show a higher yield for devices made by alcoholic antisolvents as compared to that of TFT (Figure S10, Supporting Information). The resulting power conversion efficiencies (PCEs) are highest for IPA and BuOH, averaging just below 20 %. Thereby, they exceed the average PCEs of TFT and IBA, which are below 19%. Exemplary current‐density voltage (*J*–*V*) curves are shown in Figure S11 (Supporting Information).

The very similar *V*_OC_ of the devices suggests that the changes in the vertical distribution of the PbI_2_ observed in the angular dependent GIWAXS measurements are not the cause of the improved photovoltaic performance. At the same time, it is interesting that the highest performance average was achieved for samples fabricated using IPA, which also exhibited the highest degree of preferred orientation. Considering that the improvement is associated with an enhancement in the photocurrent and not in the other solar cell parameters, it is unlikely that it originates from a change in the optoelectronic properties of the layers. Indeed, UV–Vis absorption and photoluminescence measurements (Figure S12, Supporting Information) are similar between all the measured samples. This suggests that the change in orientation mainly impacts the charge transport properties, which appears to be enhanced in the case of the highly oriented films.

To gain initial insights into the degradation behavior of the different devices, their performance was remeasured 23 days later after being stored unencapsulated in the dark in ambient air. The results are presented in Figure S13 (Supporting Information). We observe that the degradation in performance is more severe for TFT‐based devices, in comparison to that of those made using alcoholic antisolvents. This is an initial indication that the latter exhibit a slower degradation process, and considering that all other parameters in the device fabrication were kept identical, we preliminarily associate this suppression of degradation with the higher degree of orientation in the perovskite active layers. Monitoring the performance evolution of the devices under continuous illumination (Figure S14a, Supporting Information) reveals that TFT‐based devices exhibit a significantly stronger burn‐in (more than 15% of initial performance) than devices made using alcoholic antisolvents (approximately 5%). This observation is in agreement with recent reports that suggest that highly oriented films result in a superior stability under operational conditions.^[^ ^46^, ^47^, ^48^ ^]^ When exposed to thermal stress, however, the devices exhibited identical degradation dynamics (Figure S14b, Supporting Information). These results suggest that orientation plays a significant role in determining the degradation dynamics of perovskite solar cells, nevertheless, a comprehensive study of the impact of orientation on degradation mechanisms of perovskite films is a topic of future investigation and is beyond the scope of the current work.

## 3 Conclusion

To summarize, we investigated the film formation processes that govern the growth of highly oriented triple cation perovskite films fabricated by alcoholic antisolvents. By monitoring these processes by in situ GIWAXS, we uncovered the presence of a highly oriented intermediate species that templates the growth of the perovskite layers. We identify this species to be a FAI‐PbI_2_‐x·DMSO complex that is formed due to a strong interaction of the alcoholic antisolvents with the DMF host solvent of the perovskites solution, which results in its preferential extraction during the antisolvent application step. We find that films with stronger degree of orientation result in higher photovoltaic performance and stability when incorporated in solar cells, highlighting the importance of developing strategies to control the orientation of polycrystalline perovskite thin films.

## 4 Experimental Section

### Materials

Pre‐cut glass 12 × 12 mm^2^ substrates with a precoated central stripe of ITO by Psiotec Ltd. were used as a substrate for device fabrication. Perovskite precursor solution was created with PbI_2_ and PbBr_2_ from TCI, CsI from abcr, and MAI (CH_3_NH_3_I) and FAI (HC(NH_2_)_2_I) from GreatcellSolar Materials. PCBM was purchased from Lumtec and MeO‐2PACz from TCI. IBA was purchased from Alfa Aesar, EtOH from ACROS Organics and BCP, TFT and all solvents from Sigma Aldrich. The materials, solvents and solutions were stored in a dry nitrogen atmosphere except for PCBM and BCP, which were stored in ambient air. Silver pellets for thermal evaporation of the top contact were purchased from Kurt J. Lesker Company.

### Solution Preparation

MeO‐2PACz was used to form a hole‐transport layer (HTL). It was dissolved in anhydrous EtOH and the solution was sonicated for 15 min at 30 °C to 40 °C. The 1 mmol L^−1^ solution for spin coating was diluted from a 10 mmol L^−1^ stock solution.

The perovskite precursor solutions were prepared in a sequential solution method to keep a precise concentration at 1.2 mol L^−1^ of precursors for Cs_0.05_(MA_0.17_FA_0.83_)_0.95_Pb(I_0.9_Br_0.1_)_3_ in a 4:1 mixture of DMF and DMSO by volume with 1% excess of PbI_2_ and 0.25% ionic liquid 1‐butyl‐1‐methylpyrrolidinium tetrafluoroborate ([BMP]^+^[BF4]^−^) as additive. In the first step, the component salts were weighed into adequate vials. Then the inorganic salts, CsI, PbI_2_ and PbBr_2_, were dissolved in DMSO in the first case and a 4:1 mixture by volume of anhydrous DMF to DMSO in the two latter cases at 180 °C. After the salts had dissolved completely and the solutions had cooled down, the CsI and PbBr_2_ solutions were added to the PbI_2_ solution in a volume ratio of 0.05:0.15:0.85 to obtain a 1.2 mol L^−1^ inorganic stock solution of Cs_0.05_PbI_1.75_Br_0.3_ with 1% excess of PbI_2_. In a molar ratio of 0.95:1 the inorganic stock solution was added into vials with correctly weighed amounts of FAI and MAI. Then, the solution from the MAI vial was added into the FAI solution in a volume ratio of 1:5 MAI to FAI, yielding a 1.2 mol L^−1^ solution of Cs_0.05_(MA_0.17_FA_0.83_)_0.95_Pb(I_0.9_Br_0.1_)_3_ with 1% excess of PbI_2_. Finally, the appropriate amount of this solution is transferred to a vial with the ionic liquid [BMP]^+^[BF4]^−^, to yield a 0.25% concentration of the organic liquid in the resulting solution. For the electron transport layer (ETL), PCBM was dissolved in anhydrous chlorobenzene (CB) in an amber vial with a concentration of 20 mg mL^−1^. To ensure the dissolution, the mixture was stirred in a nitrogen filled glovebox (GB) overnight with a magnetic stirring bar at 70 °C. Afterwards, the solution was filtered through a 0.45 µm polytetrafluoroethylene syringe filter. As a hole‐blocking layer (HBL), BCP was deposited by means of a 0.5 mg mL^−1^ solution in anhydrous IPA. The solution was prepared by stirring overnight at 70 °C via a magnetic stirring bar under inert atmosphere.

### Device Fabrication

The devices were fabricated in an inverted architecture. Substrates were cleaned by rinsing with acetone and subsequent rinsing and 7 min sonication at 40 °C in soap water, deionized water, acetone, and isopropanol. Afterwards, the substrates were blown dry with nitrogen and exposed to an oxygen plasma for 10 min. The HTL and perovskite layer were applied in a humidity‐controlled GB. To form the HTL as a self‐assembled monolayer (SAM), 35 µL of MeO‐2PACz solution was spin‐coated statically onto the substrate at 3000 revolutions per minute (RPM) for 15 s. The samples were then annealed for 10 min at 100 °C.

Perovskite films were fabricated by applying 40 µL of precursor solution before running a two‐step spinning program. The sample was first spun at 1000 RPM for 12 s and at 5000 RPM for 28 s afterwards. Antisolvents were applied dynamically 5 s prior to the end of the fast‐spinning step in a fast manner and an amount of 150 µL. After the spinning process, the samples were annealed for 30 min at 100 °C. In the in situ GIWAXS measurements, the antisolvents were applied 10 s prior to the end of the fast‐spinning step. The ETL and HBL were fabricated in a nitrogen‐atmosphere GB. A amount of 20 µL of PCBM solution was applied dynamically after 5 s of a 30 s rotation at 2000 RPM. The samples were subsequently annealed for 10 min at 100 °C.

After cooling down, the samples were dynamically spin‐coated with 40 µL of BCP solution, applied 5 s into a 30 s spinning step at 4000 RPM. An 80 nm thick layer of 99.99% pure silver (Ag) was thermally evaporated onto the sample locally, to form top contact for the devices. To prevent harm to HBL and ETL, the deposition rate was initially set to 0.01 nm s^−1^ and increased to 0.1 nm s^−1^.

### Photovoltaic Characterization

For the PV performance measurements, an ABET TECHNOLOGIES Sun 3000 AAA solar simulator was used to illuminate the devices with simulated AM 1.5 light under ambient conditions. Currents were measured with a Keithley 2450 SMU. A NIST traceable Si reference cell was used for intensity calibration and corrected by determining the spectral mismatch between solar spectrum, reference cell, and spectral response of the device. Substrates contained eight pixels with an active device area of 1.5 × 3 mm, which were scanned with a voltage sweep from 1.2 to 0 V and back with a step size of 0.025 V and a dwell time of 0.1 s after 2 s of light soaking at 1.2 V.

### X‐Ray Diffraction

The X‐ray diffraction measurements were performed on samples containing the device structure up to the perovskite layer in ambient air. The utilized measurement system was a Bruker D8‐discover with a Lynxeye 1D detector.

### In Situ GIWAXS Characterization

The in situ GIWAXS measurements were performed at beamline P08 at PETRA III (DESY, Hamburg).^[^ ^49^ ^]^ with a photon energy of *E* = 18 keV and a Perkin Elmer XRD 1621 flat panel detector at a distance of 750 mm. The angle of incidence during in situ characterization was 0.5° to probe the bulk features of the thin films. To control the application of antisolvents during the experiments, a remote‐controlled dispensing system with an attached syringe pump was built into the measurement chamber. Both the spin‐coater and the syringe pump are integrated as devices in the beamline control software which allows for electronic synchronization of the spin‐coating procedure, antisolvent dispensing and GIWAXS measurement within an error of approximately 1 s. Diffraction intensities were calculated by integrating peak intensities over the entire peak area and applying a baseline correction. This radial integration was performed using the software ImageJ.

### Scanning Electron Microscopy

SEM measurements were performed on perovskite films on glass/ITO/MeO‐2PACz in vacuum. The prepatterned ITO stripe was used to ground the samples with silver paste to avoid sample charging. In a ZEISS GeminiSEM 500 the InLens and HE‐SE2 detectors were utilized to yield images with a 5 nm resolution with electrons of 1.5 kV landing energy.

### UV–Vis Absorption

A Jasco V‐770 Spectrophotometer was used to determine the spectral absorption of the perovskite films. The samples were glass substrates coated with perovskite and a pure glass substrate was used as reference. The spectrum was measured form 850 nm to 600 nm with a step size of 1 nm.

### Photoluminescence and PLQE

To measure PL and PLQE, the samples were fixed in the beam path of a 532 nm laser operated at 5 mW inside a calibrated Labsphere 6 inch QE sphere integration sphere and measured utilizing an Ocean Optics QE65 Pro spectrometer, following the procedure described by De Mello et al.^[^ ^50^ ^]^ During the measurement, the integration sphere was flushed with nitrogen in order to prevent oxygen or water molecules in ambient air from interacting with the perovskite surface. The samples were glass substrates coated with perovskite.

## Conflict of Interest

The authors declare no conflict of interest.

## Supporting information

Supporting Information

Supplemental Video 1

## Data Availability

The data that support the findings of this study are available from the corresponding author upon reasonable request.

## References

[1] L. Schmidt‐Mende , V. Dyakonov , S. Olthof , F. Ünlü , K. M. T. Lê , S. Mathur , A. D. Karabanov , D. C. Lupascu , L. M. Herz , A. Hinderhofer , F. Schreiber , A. Chernikov , D. A. Egger , O. Shargaieva , C. Cocchi , E. Unger , M. Saliba , M. M. Byranvand , M. Kroll , F. Nehm , K. Leo , A. Redinger , J. Höcker , T. Kirchartz , J. Warby , E. Gutierrez‐Partida , D. Neher , M. Stolterfoht , U. Würfel , M. Unmüssig , et al., APL Mater. 2021, 9, 109202.

[2] K. P. Goetz , A. D. Taylor , F. Paulus , Y. Vaynzof , Adv. Funct. Mater. 2020, 30, 1910004.

[3] M. Lai , D. Shin , L. Jibril , C. A. Mirkin , J. Am. Chem. Soc. 2022, 144, 13823. 35862285 10.1021/jacs.2c05082

[4] L. Fu , H. Li , L. Wang , R. Yin , B. Li , L. Yin , Energy Environ. Sci. 2020, 13, 4017.

[5] Z. Yang , B. H. Babu , S. Wu , T. Liu , S. Fang , Z. Xhiong , L. Han , W. Chen , Sol. RRL 2020, 4, 1900257.

[6] M. U. Rothmann , J. S. Kim , J. Borchert , K. B. Lohmann , C. M. O'Leary , A. A. Sheader , L. Clark , H. J. Snaith , M. B. Johnston , P. D. Nellist , L. M. Herz , Science 2020, 370, 6516. 10.1126/science.abb5940 33122356

[7] D. W. Quilettes , S. M. Vorpahl , S. D. Stranks , H. Nagaoka , G. E. Eperon , M. E. Ziffer , H. J. Snaith , D. S. Ginger , Science 2015, 348, 683. 25931446 10.1126/science.aaa5333

[8] Q. Sun , P. Fassl , D. Becker‐Koch , A. Bausch , S. Bai , P. E. Hopkinson , H. J. Snaith , Y. Vaynzof , Adv. Energy Mater. 2017, 7, 1700977.

[9] Q. Wang , B. Chen , Y. Liu , Y. Deng , Y. Bai , Q. Dong , J. Huang , Energy Environ. Sci. 2017, 10, 516.

[10] H. D. Kim , H. Ohkita , H. Benten , S. Ito , Adv. Mater. 2015, 28, 917. 26639125 10.1002/adma.201504144

[11] Q. An , F. Paulus , D. Becker‐Koch , C. Cho , Q. Sun , A. Weu , S. Bitton , N. Tessler , Y. Vaynzof , Matter 2021, 4, P4051.

[12] B. Lee , T. Hwang , S. Lee , B. Shin , B. Park , Sci. Rep. 2019, 9, 4803. 30886329 10.1038/s41598-019-41328-5 PMC6423327

[13] J. V. Patil , S. S. Mali , C. K. Hong , Nanoscale 2019, 11, 21824. 31693036 10.1039/c9nr07377a

[14] W. Xu , G. Lei , C. Tao , J. Zhang , X. Liu , X. Xu , W.‐Y. Lai , F. Gao , W. Huang , Adv. Funct. Mater. 2018, 28, 1802320.

[15] Q. An , F. Paulus , Y. Vaynzof , ACS Appl. Energy Mater. 2021, 4, 2990. 10.1021/acsaem.5c03034 PMC12933516 41756055

[16] C. Bi , Q. Wang , Y. Shao , Y. Yuan , Z. Xiao , J. Huang , Nat. Commun. 2015, 6, 7747. 26190275 10.1038/ncomms8747 PMC4518278

[17] M. Degani , Q. An , M. Albaladejo‐Siguan , Y. J. Hofstetter , C. Cho , F. Paulus , G. Grancini , Y. Vaynzof , Sci. Adv. 2021, 7, eabj7930. 34851671 10.1126/sciadv.abj7930 PMC8635431

[18] C. Jiang , P. Zheng , J. Appl. Phys. 2018, 123, 083105.

[19] R. Wang , J. Xue , L. Meng , J.‐W. Lee , Z. Zhao , P. Sun , L. Cai , T. Huang , Z. Wang , Z.‐K. Wang , Y. Duan , J. L. Yang , S. Tan , Y. Yuan , Y. Huang , Y. Yang , Joule 2019, 3, 1464.

[20] L. A. Muscarella , E. M. Hutter , S. Sanchez , C. D. Dieleman , T. J. Savenije , A. Hagfeldt , M. Saliba , B. Ehrler , J. Phys. Chem. Lett. 2019, 10, 6010. 31542932 10.1021/acs.jpclett.9b02757 PMC6801854

[21] S. Chen , X. Xiao , B. Chen , L. L. Kelly , J. Zhao , Y. Lin , M. F. Toney , J. Huang , Sci. Adv. 2021, 7, abb2412.10.1126/sciadv.abb241233523938

[22] Y. Xia , X. Ran , Y. Chen , Q. Li , N. Jiang , C. Li , Y. Pan , T. Li , J. Wang , W. Huang , J. Mater. Chem. A 2017, 5, 3193.

[23] K. Wang , M.‐C. Tang , H. X. Darng , R. Munir , D. Barrit , M. De Bastiani , E. Aydin , D.‐M. Smilgies , S. De Wolf , A. Amassian , Adv. Mater. 2019, 31, 1808357. 10.1002/adma.201808357 31206857

[24] W. Xiang , J. Zhang , S. Liu , S. Albrecht , A. Hagfeldt , Z. Wang , Joule 2022, 6, 315.

[25] M. Qin , P. F. Chan , X. Lu , Adv. Mater. 2021, 33, 2105290. 10.1002/adma.202105290 34605066

[26] N. Arora , A. Greco , S. Meloni , A. Hinderhofer , A. Mattoni , U. Rothlisberger , J. Hagenlocher , C. Caddeo , S. M. Zakeeruddin , F. Schreiber , M. Graetzel , R. H. Friend , M. I. Dar , Commun. Mater. 2022, 3, 22.

[27] A. Greco , A. Hinderhofer , M. Dar , A. Ibrahim , N. Arora , J. Hagenlocher , A. Chumakov , M. Grätzel , F. Schreiber , J. Phys. Chem. Lett. 2018, 9, 6750. 30400741 10.1021/acs.jpclett.8b02916

[28] M. Qin , K. Tse , T.‐K. Lau , Y. Li , C.‐J. Su , G. Yang , J. Chen , J. Zhu , U.‐S. Jeng , G. Li , H. Chen , X. Lu , Adv. Mater. 2019, 31, 1901284. 10.1002/adma.201901284 31038236

[29] T. Bu , J. Li , H. Li , C. Tian , J. Su , G. Tong , L. K. Ono , C. Wang , Z. Lin , N. Chai , X.‐L. Zhang , J. Chang , J. Lu , J. Zhong , W. Huang , Y. Qi , Y.‐B. Cheng , F. Huang , Science 2021, 372, 1327. 34140385 10.1126/science.abh1035

[30] A. D. Taylor , Q. Sun , K. P. Goetz , Q. An , T. Schramm , Y. Hofstetter , M. Litterst , F. Paulus , Y. Vaynzof , Nat. Commun. 2021, 12, 1878. 33767163 10.1038/s41467-021-22049-8 PMC7994557

[31] X. Wang , Z. Han , F. Gao , C. Luo , Q. Zhao , Sol. RRL 2022, 6, 2100973.

[32] Q. Hu , L. Zhao , J. Wu , K. Gao , D. Luo , Y. Jiang , Z. Zhang , C. Zhu , E. Schaible , A. Hexemer , C. Wang , Y. Liu , W. Zhang , M. Grätzel , F. Liu , T. P. Russel , R. Zhu , Q. Gong , Nat. Commun. 2017, 8, 15688. 28635947 10.1038/ncomms15688 PMC5482054

[33] P. Gratia , I. Zimmermann , P. Schouwink , J.‐H. Yum , J.‐N. Audinot , K. Sivula , T. Wirtz , M. K. Nazeeruddin , ACS Energy Lett. 2017, 2, 2686.

[34] L. Chen , Y.‐Y. Tan , Z.‐X. Chen , T. Wang , S. Hu , Z.‐A. Nan , L.‐Q. Xie , Y. Hui , J.‐X. Huang , C. Zhan , S.‐H. Wang , J.‐Z. Zhou , J.‐W. Yan , B.‐W. Mao , Z.‐Q. Tian , J. Am. Chem. Soc. 2019, 141, 1665. 30608680 10.1021/jacs.8b11610

[35] J. Cao , X. Jing , J. Yan , C. Hu , R. Chen , J. Yin , J. Li , N. Zheng , J. Am. Chem. Soc. 2016, 138, 9919. 27427774 10.1021/jacs.6b04924

[36] Z. Hu , Q. An , H. Xiang , L. Aigouy , B. Sun , Y. Vaynzof , Z. Chen , ACS Appl. Mater. Interfaces 2020, 12, 54824. 33226765 10.1021/acsami.0c17258

[37] A. Kogo , T. Miyadera , M. Chikamatsu , ACS Appl. Mater. Interfaces 2019, 11, 38683. 31553161 10.1021/acsami.9b12193

[38] O. Telschow , M. Albaladejo‐Siguan , L. Merten , A. D. Taylor , K. P. Goetz , T. Schramm , O. V. Konovalov , M. Jankowski , A. Hinderhofer , F. Paulus , F. Schreiber , Y. Vaynzof , J. Mater. Chem. A 2022, 10, 19743.

[39] Y. Rong , Z. Tang , Y. Zhao , X. Zhong , S. Venkatesan , H. Graham , M. Patton , Y. Jing , A. M. Guloy , Y. Yao , Nanoscale 2015, 7, 10595. 26037081 10.1039/c5nr02866c

[40] F. Hao , C. C. Stoumpos , Z. Liu , R. P. H. Chang , M. G. Kanatzidis , J. Am. Chem. Soc. 2014, 136, 16411. 25374278 10.1021/ja509245x

[41] A. Wakamiya , M. Endo , T. Sasamori , N. Tokitoh , Y. Ogomi , S. Hayase , Y. Murata , Chem. Lett. 2014, 43, 711.

[42] Y. Ren , N. Zhang , Q. Wang , J. Zhu , C. Li , Sci. China Mater. 2020, 63, 1015.

[43] C. Hansen , The Three Dimensional Solubility Parameter and Solvent Diffusion Coefficient and Their Importance in Surface Coating Formulation, Danish Technical Press, Copenhagen 1967.

[44] Q. An , L. Vieler , K. P. Goetz , O. Telschow , Y. J. Hofstetter , R. Buschbeck , A. D. Taylor , Y. Vaynzof , Adv. Energy Sustainablility Res. 2021, 2, 2100061.

[45] S. Cacovich , G. Vidon , M. Degani , M. Legrand , L. Gouda , J.‐B. Puel , Y. Vaynzof , J.‐F. Guillemoles , D. Ory , G. Grancini , Nat. Commun. 2022, 13, 2868. 35606374 10.1038/s41467-022-30426-0 PMC9126963

[46] C. Ma , M.‐C. Kang , S.‐H. Lee , S. J. Kwon , H.‐W. Cha , C.‐W. Yang , N.‐G. Park , Joule 2022, 11, 2626.

[47] C. Ma , F. T. Eickemeyer , S.‐H. Lee , D.‐H. Kang , S. J. Kwon , M. Graetzel , N.‐G. Park , Science 2023, 379, 173. 36634188 10.1126/science.adf3349

[48] D. P. McMeekin , P. Holzhey , S. O. Fürer , S. P. Harvey , L. T. Schelhas , J. M. Ball , S. Mahesh , S. Seo , N. Hawkins , J. Lu , M. B. Johnston , J. J. Berry , U. Bach , H. J. Snaith , Nat. Mater. 2023, 22, 73. 36456873 10.1038/s41563-022-01399-8

[49] O. H. Seeck , C. Deiter , K. Pflaum , F. Bertam , A. Beerlink , H. Franz , J. Horbach , H. Schulte‐Schrepping , B. M. Murphy , M. Greve , O. Magnussen , J. Synchrotron Radiat. 2012, 19, 30. 22186641 10.1107/S0909049511047236

[50] J. C. de Mello , H. F. Wittmann , R. H. Friend , Adv. Mater. 1997, 9, 230.

