# Supplementary material for: Elucidating Structure Formation in Highly Oriented Triple Cation Perovskite Films
Source: Adv Sci (Weinh). 2023 Apr 20;10(17):2206325. doi: 10.1002/advs.202206325 (PMC10265059; doi:10.1002/advs.202206325)
Supplement: Supplementary file 1 — Supporting Information [file ADVS-10-2206325-s002.pdf]

ADVANCED SCIENCE Open Access

Supporting Information

for *Adv. Sci.*, DOI 10.1002/advs.202206325

Elucidating Structure Formation in Highly Oriented Triple Cation Perovskite Films

*Oscar Telschow, Niels Scheffczyk, Alexander Hinderhofer, Lena Merten, Ekaterina Kneschaurek, Florian Bertram, Qi Zhou, Markus Löffler, Frank Schreiber, Fabian Paulus and Yana Vaynzof**

Supporting Information

Elucidating Structure Formation in Highly Oriented Triple Cation Perovskite Films

*Oscar Telschow, Niels Scheffczyk, Alexander Hinderhofer, Lena Merten, Ekaterina Kneschaurek, Florian Bertram, Qi Zhou, Markus Löffler, Frank Schreiber, Fabian Paulus and Yana Vaynzof**

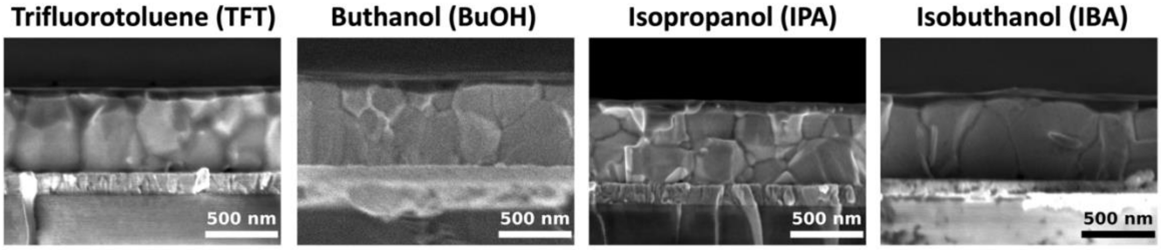

Figure S1: Cross-sectional SEM images of perovskite films fabricated using the different antisolvents. Please note that the films are coated with PCBM and BCP following the structure of the photovoltaic devices.

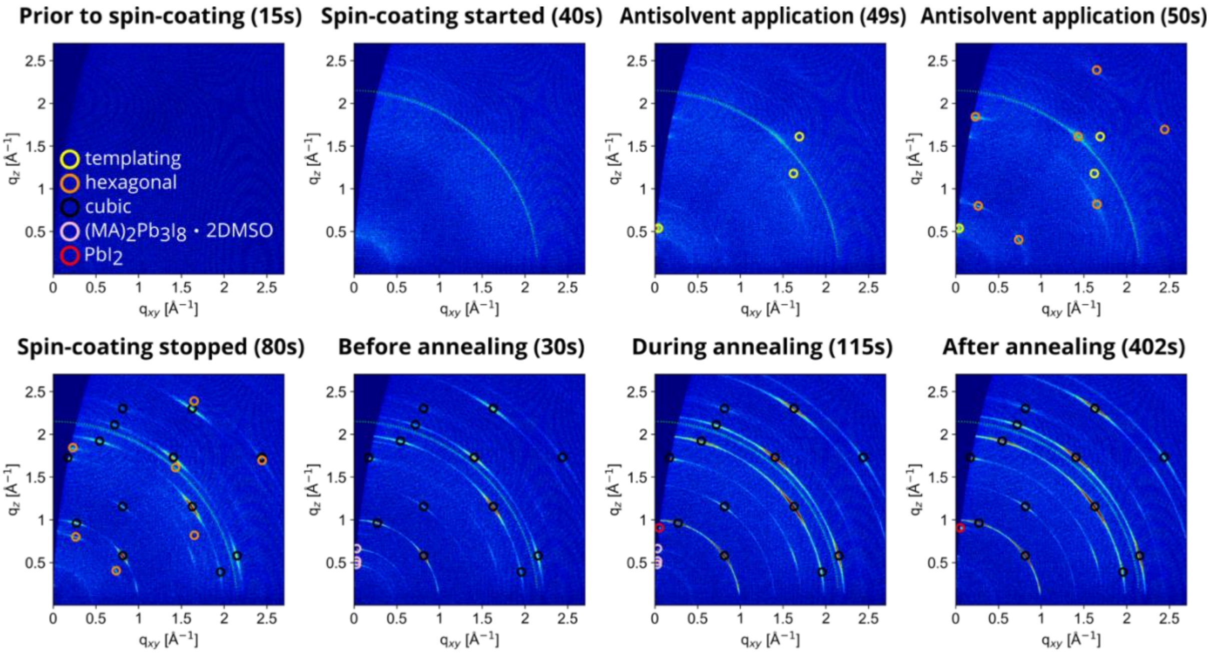

Figure S2: GIWAXS maps taken during different time points during film formation using IBA as an antisolvent.

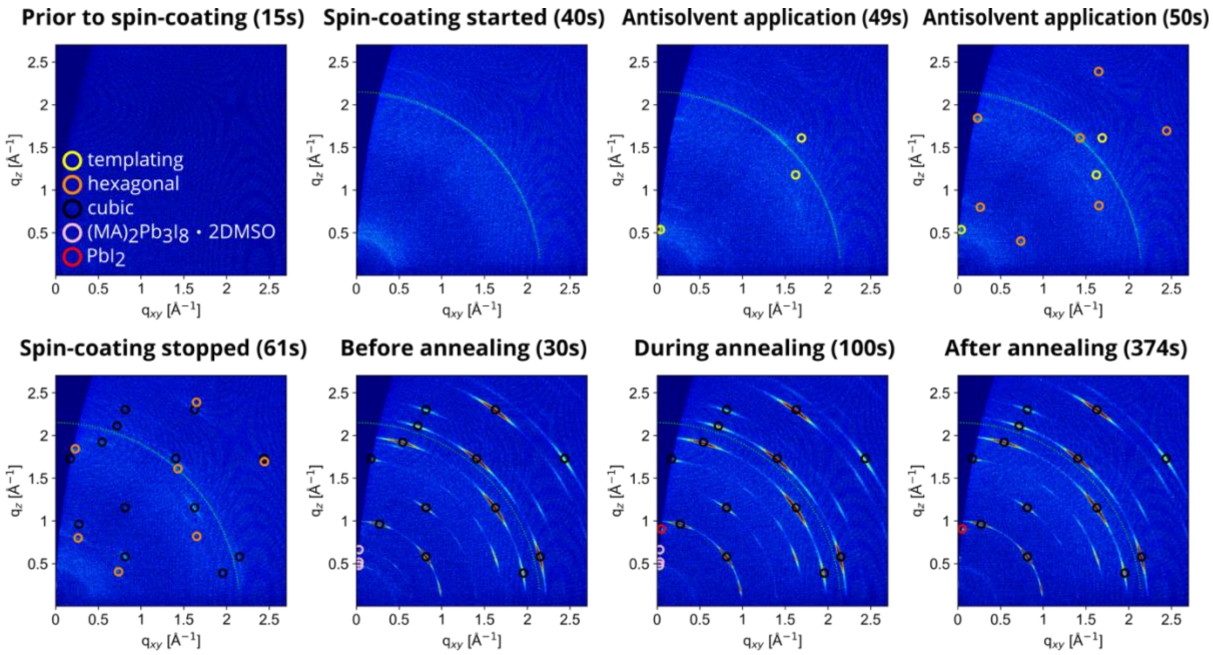

Figure S3: GIWAXS maps taken during different time points during film formation using BuOH as an antisolvent.

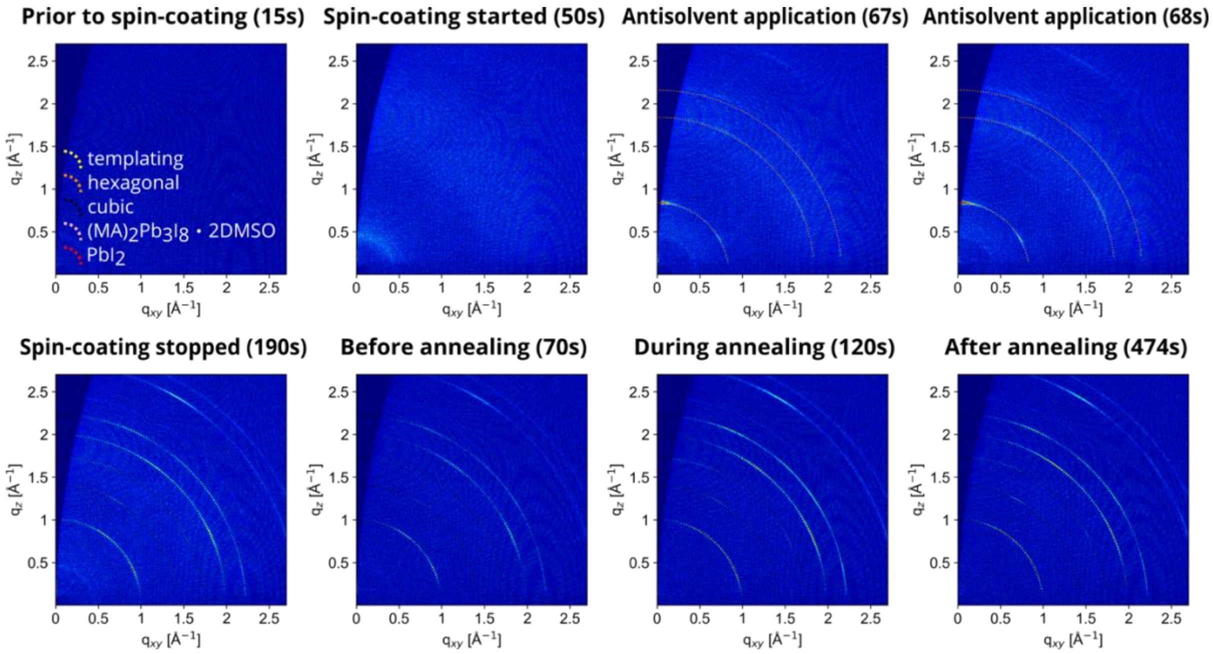

Figure S4: GIWAXS maps taken during different time points during film formation using TFT as an antisolvent.

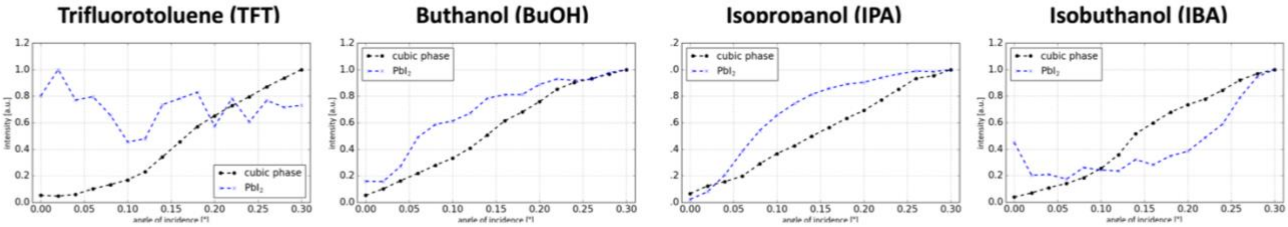

Figure S5: Intensity evolution of cubic perovskite and PbI_2 as a function of angle for fully fabricated films made using different antisolvents.

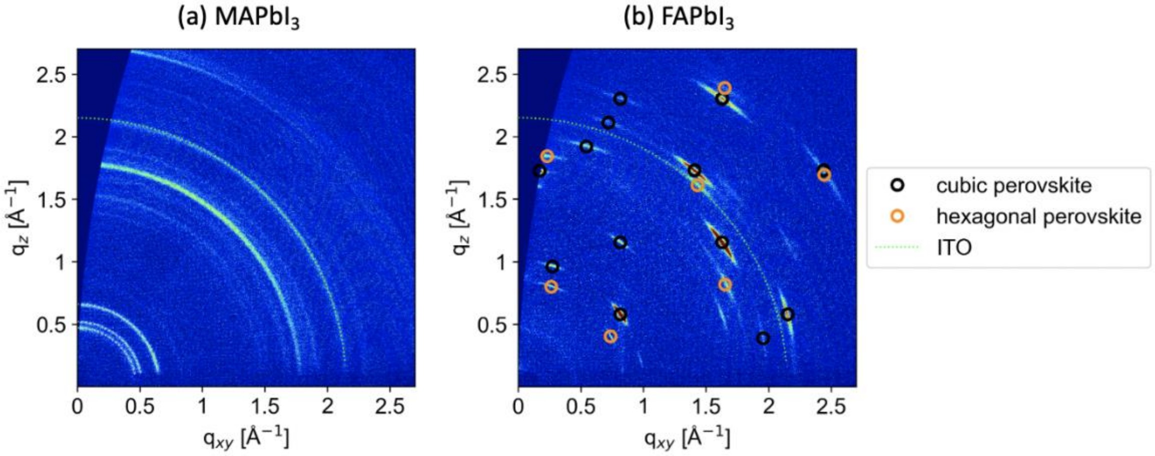

Figure S6: GIWAXS data after spin coating (a) $MAPbI_3$ and (b) $FAPbI_3$ films made using IPA as an antisolvent.

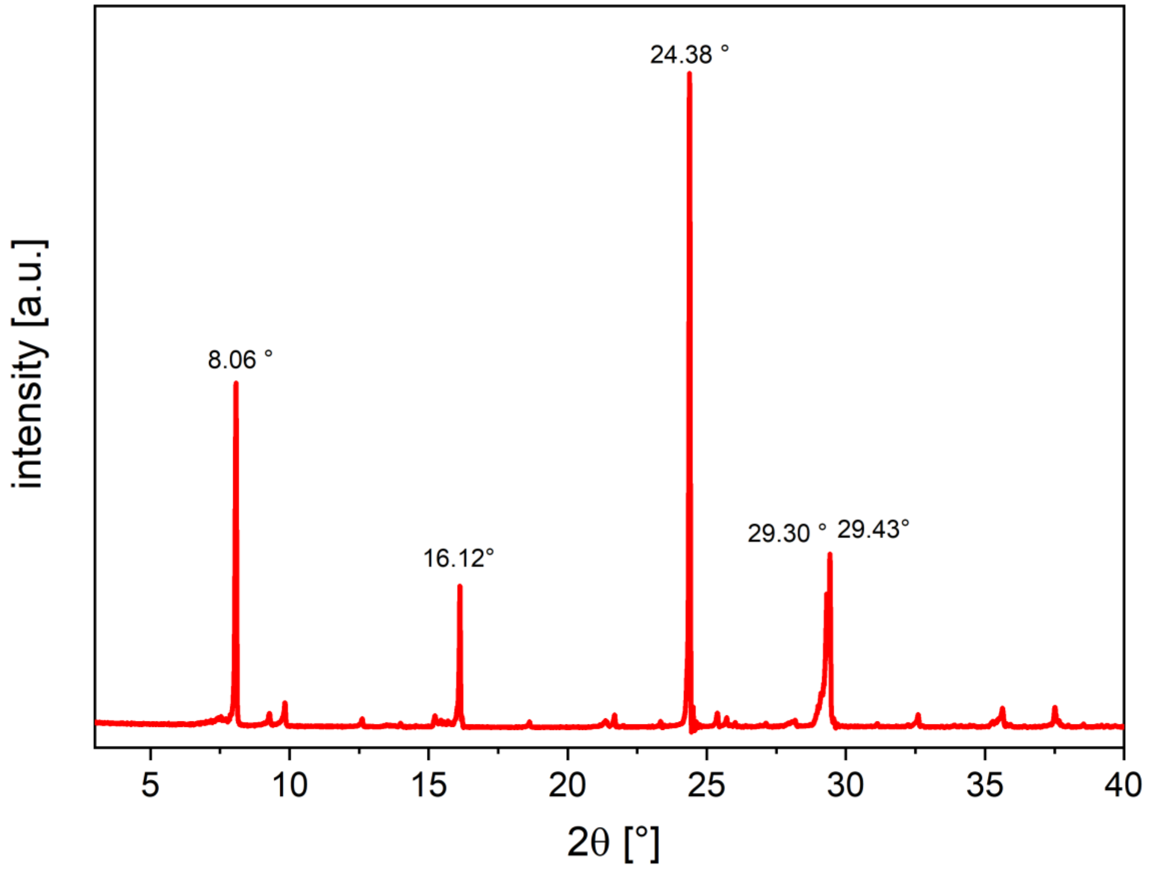

Figure S7: XRD pattern of a crystalline film obtained by drop-casting a highly concentrated solution of FAI and PbI_2 in a molar 1:1 ratio in pure anhydrous DMSO after gentle drying at 60°C.

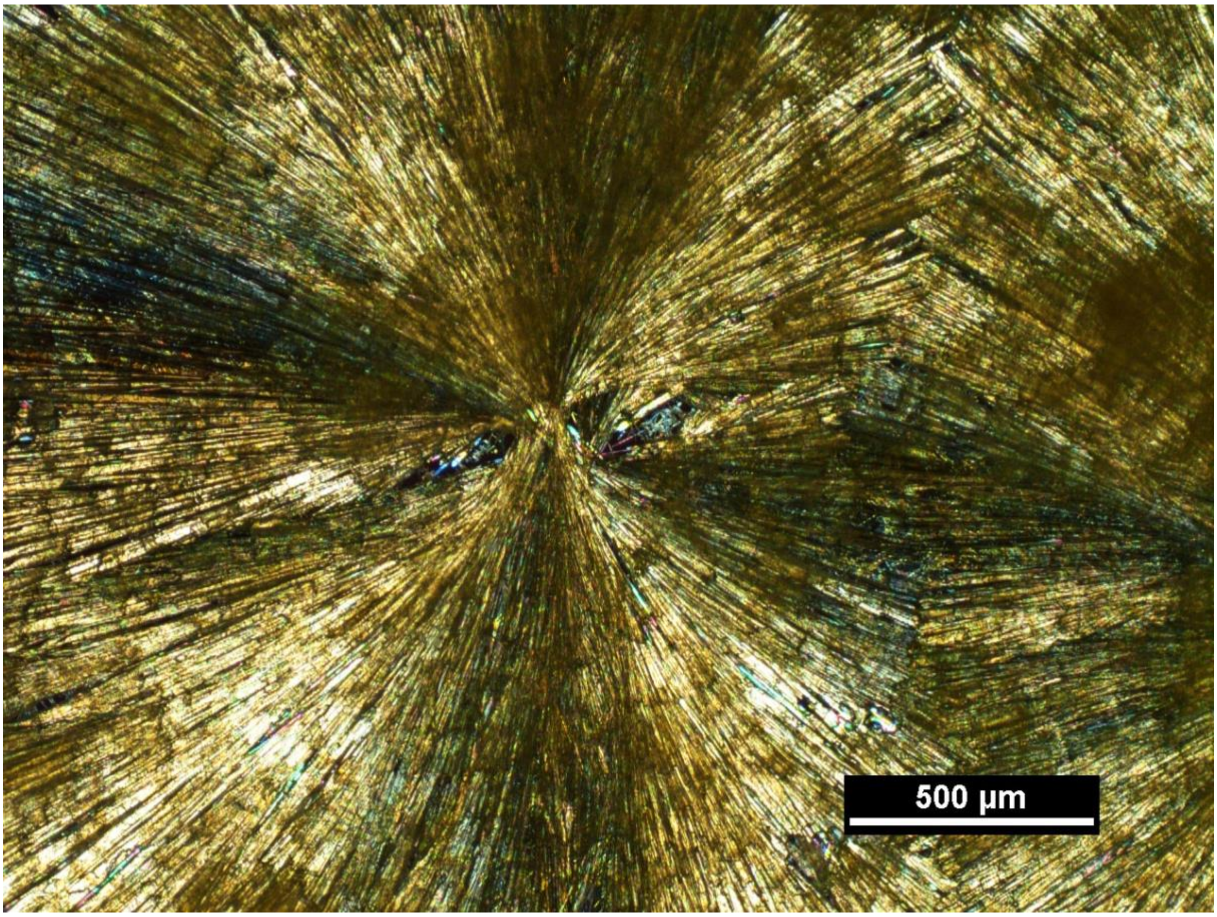

Figure S8: Optical microscopy image of a crystalline film obtained by drop-casting a highly concentrated solution of FAI and PbI_2 in a molar 1:1 ratio in pure anhydrous DMSO after gentle drying at 60°C.

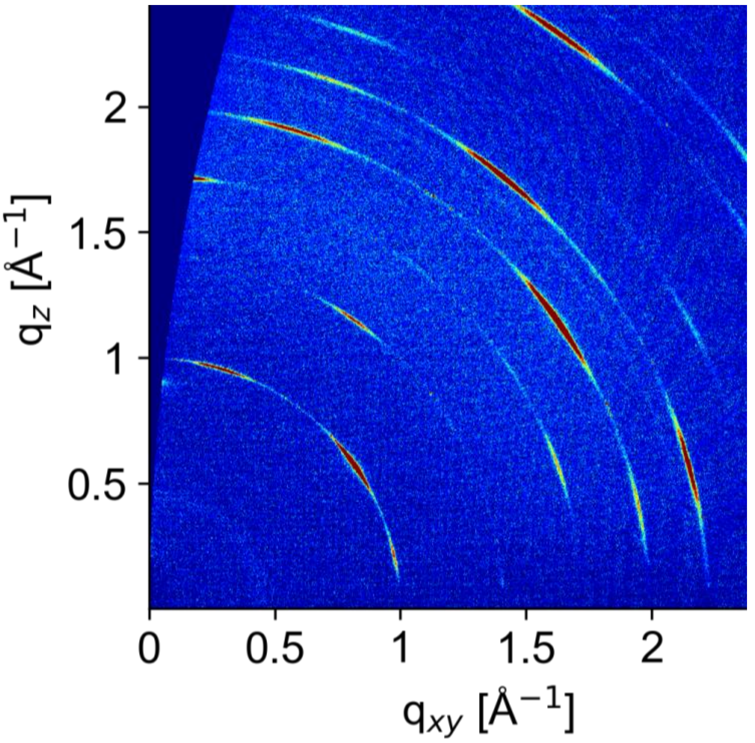

Figure S9: GIWAXS data for a perovskite samples fabricated using a 1:1 TFT:IPA antisolvent mixture.

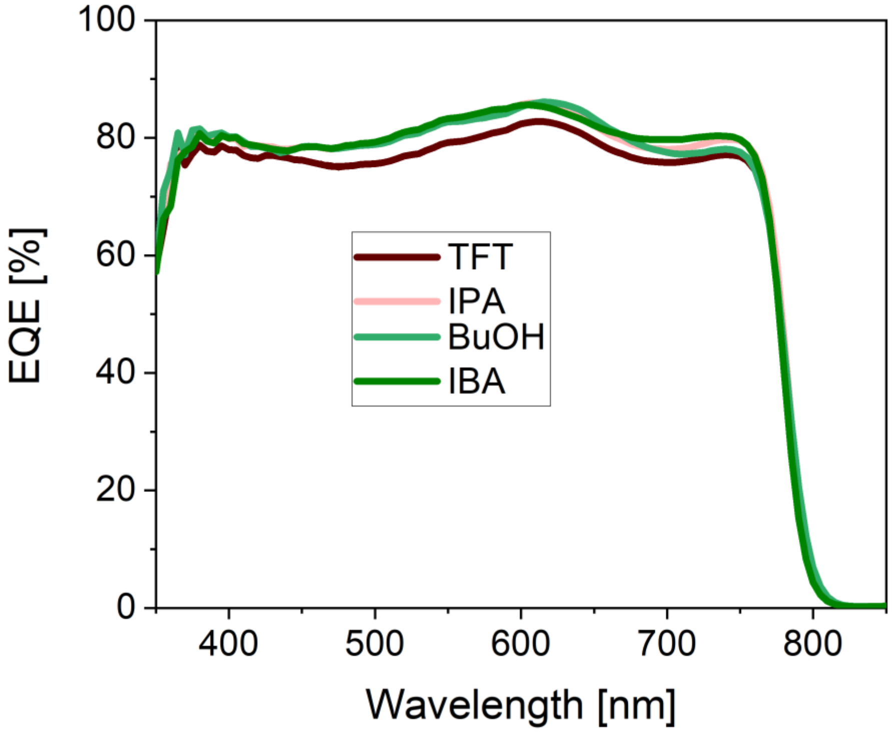

Figure S10: External quantum efficiency (EQE) measurements of devices fabricated using different antisolvents.

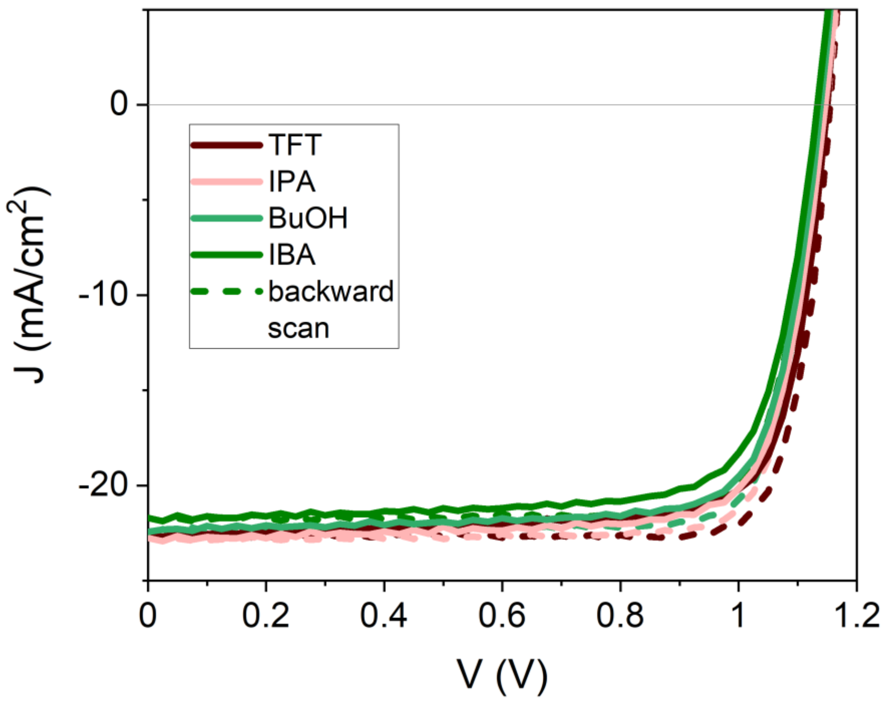

Figure S11: J-V curves of representative devices made using the different antisolvents.

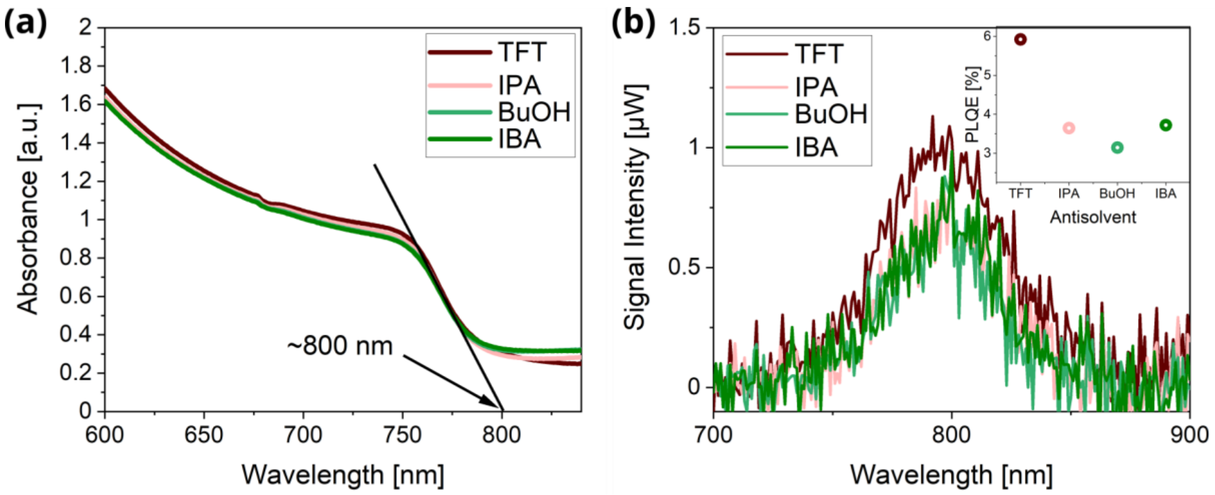

Figure S12: (a) UV-vis and (b) PL spectra of perovskite films fabricated using different antisolvents.

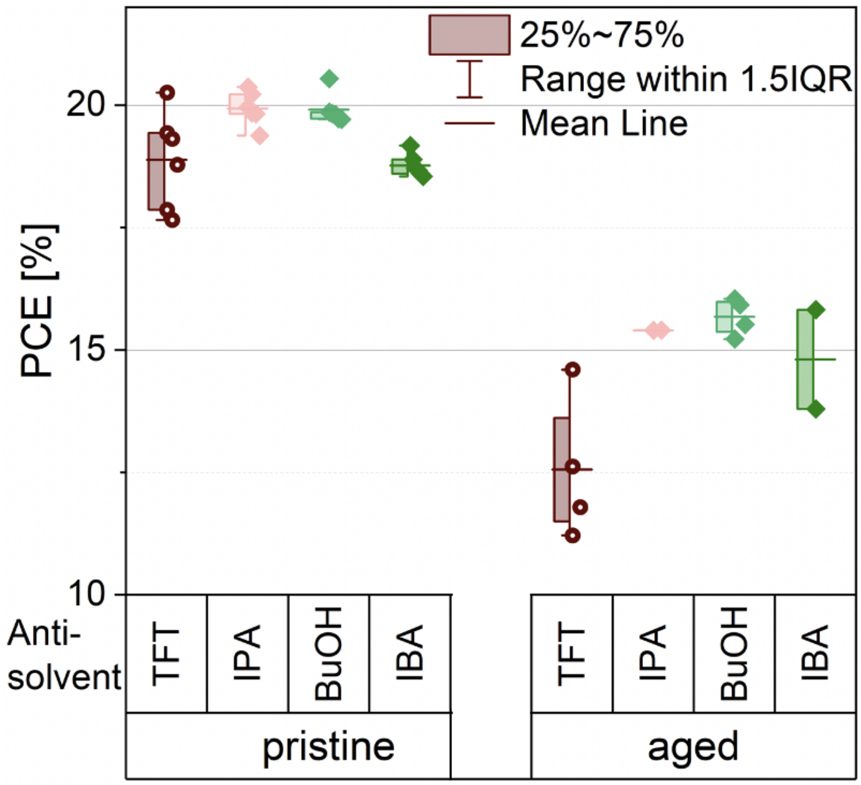

Figure S13: Performance of photovoltaic devices fabricated using different antisolvents upon storage without encapsulation in the dark in ambient air for 23 days.

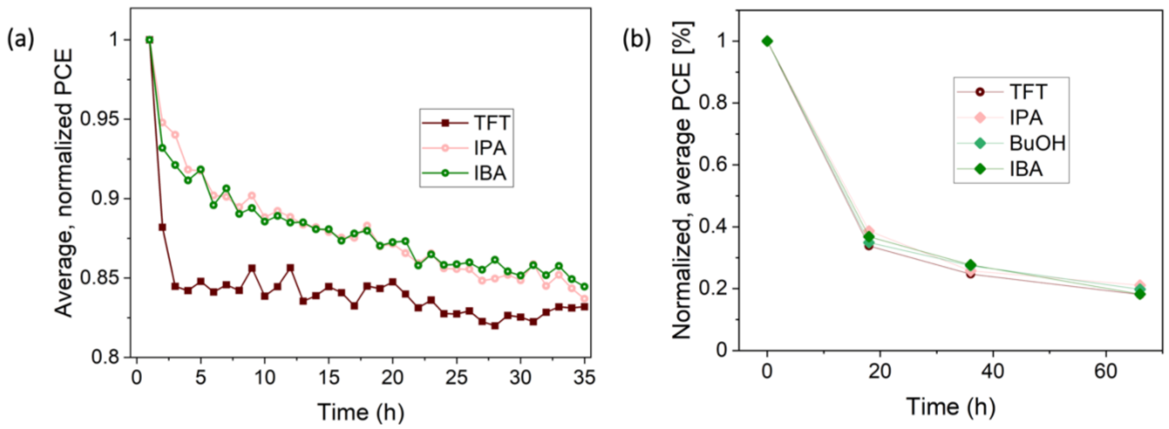

Figure S14: Performance evolution of unencapsulated photovoltaic devices fabricated using different antisolvents upon (a) continuous illumination at 1 Sun stored in N_2 and measured in air and (b) annealing at 85 °C in air.

Supplementary Note 1:

Video files of the GIWAXS maps recorded during the spin-coating and annealing of samples fabricated by the four different antisolvents are included in the supporting information section. High resolution versions of these videos are available from the authors upon request.
